# Virtual vs Online: Insight From Medical Students. Comment on “Effectiveness of Virtual Medical Teaching During the COVID-19 Crisis: Systematic Review”

**DOI:** 10.2196/27020

**Published:** 2021-05-14

**Authors:** Shahil Kaini, Lucinda Zahrah Motie

**Affiliations:** 1 University College London London United Kingdom

**Keywords:** virtual teaching, medical student, medical education, COVID-19, review, search term, virus, pandemic, quarantine

We read with great interest the review by Wilcha [1], which discussed current literature on the effectiveness of virtual medical teaching during COVID-19. The conclusion suggested that despite various disadvantages, virtual teaching effectively enabled medical education to continue during the peak of the COVID-19 pandemic. As fifth-year medical students currently studying at University College London, we have first-hand experience in virtual teaching and recognize its importance within medical education in the future.

We commend Wilcha for undertaking a systematic review in a newly emerging area of medical education. Virtual teaching appears likely to remain a part of medical education going forward; therefore, we welcome any attempts to review existing research to outline advantages, disadvantages, and recommendations. As acknowledged by Wilcha, researching an emerging area means the literature available is restricted. However, we would like to question the literature search conducted. Wilcha used the key term “virtual,” when searching PubMed and Google Scholar, but we believe this limited the search. The search could have been expanded by using “online,” a term synonymous with “virtual.” We repeated the original search, replacing the term “virtual” with “online.” This yielded 108 articles on PubMed compared to the 92 as originally reported. We then put these articles through the inclusion and exclusion criteria outlined in the *Methods* section of the review. This left us with 7 articles, all of which were published in a peer-reviewed scientific journal, relevant to the objectives of the study, and conducted between February and June 2020.

Some of these missed articles raise interesting points. For example, Wilcha discusses how technological difficulties are a major disadvantage of virtual teaching. However, Nik-Ahmad-Ziki et al [2] raise excellent points regarding this topic that were not discussed in the review. They outline the psychological impact technological difficulties can have on students, leaving them discouraged from joining sessions and demotivated. Interestingly, technological difficulties were rare for clinical teachers, who still had to go to the hospital during the day and so had access to excellent facilities and internet coverage.

Research on virtual teaching has become very important due to the recent changes enforced by the COVD-19 pandemic. We commend Wilcha for conducting this systematic review, but we believe the initial literature search was too limited. Adjusting the search terms would have provided more literature to review and more points to discuss. Furthermore, it would have helped address certain limitations. Many of the studies discussed by Wilcha had small sample sizes, which decreases the reliability of the findings. Had “online” been used as a key term, the study by Singh et al [3] would have been included. This study had a large sample size of 208 students and presented interesting findings as many students thought physical classes were better than virtual classes. Going forward, repeating this systematic review would be useful as a considerable amount of research has occurred on this topic since this review was originally conducted.
